# Can smartphone wireless ECGs be used to accurately assess ECG intervals in pediatrics? A comparison of mobile health monitoring to standard 12-lead ECG

**DOI:** 10.1371/journal.pone.0204403

**Published:** 2018-09-27

**Authors:** Melanie R. F. Gropler, Aarti S. Dalal, George F. Van Hare, Jennifer N. Avari Silva

**Affiliations:** Department of Pediatrics, Division of Pediatric Cardiology, Washington University School of Medicine, Saint Louis, MO, United States of America; Duke-NUS Medical School, SINGAPORE

## Abstract

**Background:**

Arrhythmias in children are often paroxysmal, complicating the ability to capture the abnormal rhythm on routine ECG during an outpatient visit. The Alivecor Kardia Mobile (KM) device is a wireless mobile health (mHealth) device that generates a single lead ECG tracing with a FDA-approved algorithm for detection of atrial fibrillation in adults.

**Objective:**

The goal of this study is to assess the accuracy of interval measurements on KM tracings by directly comparing to standard 12-lead ECGs in pediatric patients.

**Methods:**

This single center, prospective study enrolled pediatric outpatients, age <18 years presenting for cardiology clinic visits, into 3 groups based on age: 0–5 years, 6–10 years, and 11–18 years. Patients were excluded if 12-lead ECG was not ordered during the visit. Each enrolled subject underwent standard 12-lead ECG followed by 30-second KM tracing. ECG parameters were batch read by 2 blinded pediatric electrophysiologists.

**Results:**

Thirty patients were recruited with 10 patients/group. Structural heart disease and/or conduction abnormality was present in 20 patients (67%). Majority of tracings (27/30, 90%) were of diagnostic quality on first attempt. Overall, the ΔPR was 15.2±10.8ms (r = 0.86), ΔQRS was 9.6±8ms (r = 0.86), and ΔQTc was 15.6±12.7ms (r = 0.83). There were 9 patients with ΔQTc measurements >20ms with 4/9 (44%) having a conduction disorder and 2/9 (22%) having marked sinus arrhythmia. Bland-Altman method of agreement demonstrated strong agreement for QRSd and QTc. The AF algorithm reported 4/30 (13%) false positive "possible AF" diagnoses (rhythm over-read on KM demonstrated n = 3 marked sinus arrhythmia, n = 1 sinus rhythm with aberrated PACs) resulting in a specificity of 87%.

**Conclusion:**

The Alivecor Kardia device produces accurate single lead ECG tracings in both healthy children and children with cardiac disease or rhythm abnormalities across the pediatric spectrum. This mHealth application provides an accurate, non-invasive, real-time approach for ambulatory ECG monitoring in children and adolescents.

## Introduction

Cardiac arrhythmias are the most frequently encountered complication in children and adults with history of congenital heart disease [1]. In addition to this population of children with congenital cardiac malformations, the American Heart Association’s Task Force on Children and Youth (1993) reported that approximately 30,000 children develop a cardiac arrhythmia or are born with an abnormality of cardiac conduction [2]. Arrhythmias in children are often paroxysmal, complicating the ability to capture the abnormal rhythm on routine electrocardiogram during an outpatient visit. Current practice utilizes 24-hour ambulatory monitors, external event monitors, or implantable loop recorders (ILR) to evaluate children with cardiac arrhythmias. Disadvantages of these methods include cost, invasiveness (surgical placement of ILR), sensitivity to lead adhesive, and inability to provide real-time access to transmitted ECG tracings.

Smartphones are increasingly prevalent in today’s society and their ubiquity among children and adolescents is no exception. Data suggest that 85% of teens and caregivers utilize smartphones and 95% of teens are now “online.” [3] Advancements in wireless technology and digital communication have led to the ability to perform health monitoring through mobile technology. The AliveCor Kardia heart monitor (Smartphone monitor, AliveCor, San Francisco, CA, USA) provides a single-lead rhythm strip comparable to lead 1 on standard 12-lead ECG machines using a smartphone. The Alivecor Kardia mobile device is commercially available as a direct to consumer product and does have FDA approval for use in adults with atrial fibrillation. To date, there is no approved FDA indication for use in the pediatric population. Haberman et al demonstrated that this method accurately detects baseline intervals, atrial rate, and rhythm in diverse populations [4]. The monitor has also been studied in children. Nguyen et al reported the ability of the Kardia device to generate diagnostic quality tracings, highlighting the potential to improve outpatient management of pediatric cardiac arrhythmias [5]. In this study, we aim to study the accuracy and usability of the Kardia monitor (KM) as compared to standard 12-lead ECG in a pediatric outpatient setting. This is the first study to directly compare these two modalities in pediatric patients.

## Materials and methods

This prospective study received full approval from the Washington University in St. Louis’s Institutional Review Board. Patients scheduled in the outpatient cardiology clinic were recruited if they met the following inclusion criteria: 1) age < 18 years and 2) standard 12-lead ECG ordered as part of routine visit testing. Written consent was obtained from the parent/guardian and assent was also obtained for those patients ≥12 years of age.

After consent was completed, patients and caregivers were instructed on how to use the KM device. Patients were instructed to place 2 fingers of each hand on the respective device electrodes. A 30-second lead 1 tracing was recorded using the KM iPhone application. KM tracings were performed either before or after 12-lead ECG tracings, not concurrently. Both KM tracings and 12-lead ECG were saved and de-identified for offline analysis. Chart review was also performed to obtain patient demographic data, cardiac history, and ECG indication. Recruitment was continued until 30 patients were enrolled with 10 patients in each of the following groups: 0–5 years (group A), 6–10 years (group B), 11–18 years (group C).

For the older patients in group A, the most successful method to obtain KM tracings was to have the patient sit on the caregiver’s lap facing outward, with the caregiver cupping their hands around the child who would be holding on the electrodes, as if holding a book. For the infants in group A, the most ideal positioning was to set their fingers on top of the KM electrode either while they were in their caregiver’s lap or when they were lying on the exam table. Obtaining a tracing in infants and toddlers frequently required two people to coordinate the recording and appropriate finger positioning.

KM tracings and 12-lead ECGs were batch read by two board-certified pediatric electrophysiologists (EP). ECG tracings from both the KM and 12-lead ECG were provided to reviewers in both electronic and paper format for ease of reading and calculation. Heart rhythm, heart rate, and interval measurements (RR, PR, QRS, QT, and QTc) were recorded. QTc was calculated using Bazett’s formula [6]. All measurements from each EP for both the KM and 12-lead ECG tracings were averaged to account for inter-user variability. Perfect agreement between modalities was defined as an absolute difference of <20 msec [7].

### Statistical analysis

Summary data are presented as frequency with percentage. Descriptive statistics are presented as means with range. The Bland-Altman method as well as correlation analysis was used to compare interval agreement. Repeat measure analysis of variance (ANOVA) and student’s t-test were used to compare differences in bias between age groups when appropriate. Statistical significance was defines as a *p*-value ≤ 0.05.

## Results

### Demographics

**Fig 1:**
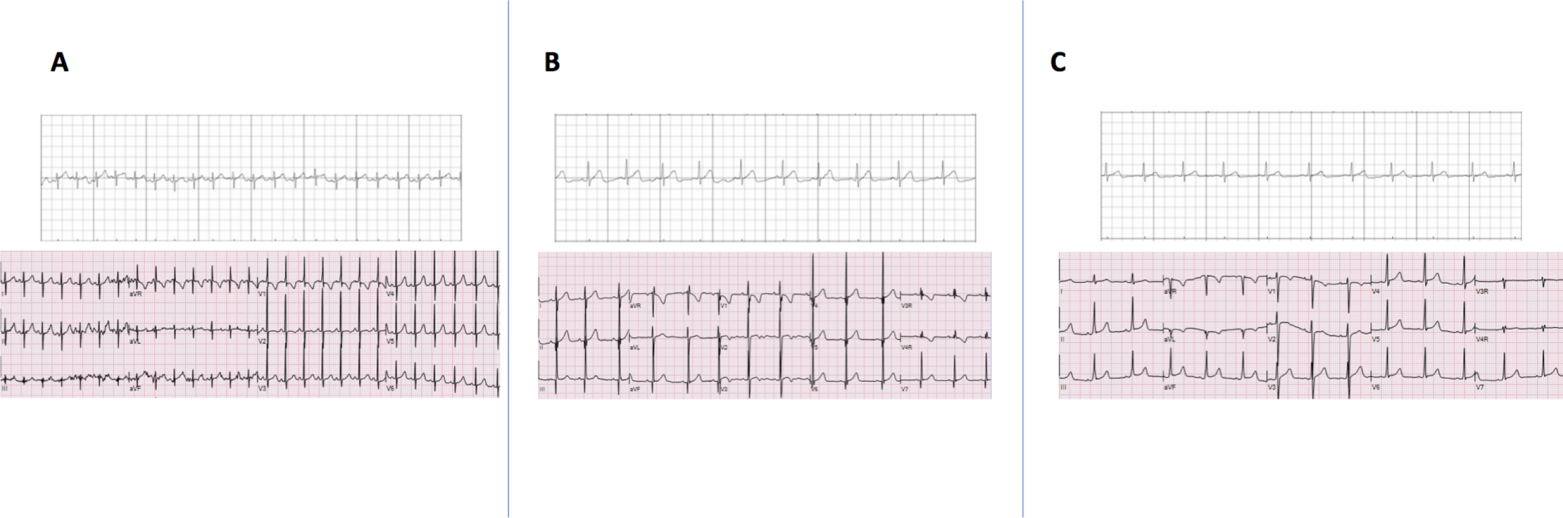
Side-by-side examples of serially acquired KM tracings and 12-lead ECGs exemplifying diagnostic quality of the KM for a patient in group A (panel A), group B (panel B), and group C (panel C).

Thirty patients were recruited with 10 patients/group with all patients in sinus rhythm at time of data collection. Demographic data are presented in Table 1. The majority of tracings, 27/30 (90%), were diagnostic quality on first attempt with 3/30 (10%) requiring a second tracing to obtain diagnostic data (See Fig 1). Specifically, one patient in group C and two patients in group A needed to repeat the recording when the first recording terminated at < 30 seconds for inadequate contact with the electrode in the former and for premature movement of fingers off of the electrode for the latter. Congenital heart disease and/or conduction abnormalities were present in 20/30 patients (67%).

**Table 1:** Demographic data. Patient demographic data is shown with descriptive statistical data. Abbreviations: CoA = coarctation of the aorta, BAV = bicuspid aortic valve, VSD = ventricular septal defect, ASD = atrial septal defect, SVC = superior vena cava, PV = pulmonary valve, JET = junctional ectopic tachycardia, HB = heart block, LQTS = long QT syndrome, SVT = supraventricular tachycardia, WPW = Wolff-Parkinson-White syndrome.

Patient characteristics	N (range/%)
Mean age (years)	8.2 (14 days-17 years)
Male	14 (47%)
Mean weight (kg)	38 (3.9–110.1)
Mean height (cm)	119 (50.8–172.7)
Cardiac Diagnosis(es)	
*Congenital Heart Disease*	7 (23%)
CoA, BAV	1
VSD	1
ASD	2
Left SV with unroofed coronary sinus	1
Congenital PV Stenosis	1
BAV	1
*Arrhythmia*	10 (33%)
JET	1
Second degree HB	1
WPW	3
PVC	1
Inappropriate sinus tachycardia	2
LQTS Type 3	1
Neonatal SVT	1
*Congenital Heart Disease + arrhythmia*	3 (10%)
ASD + SVT	1
AVC defect + WPW	1
Shone’s Complex + CHB + pacemaker	1

### Baseline intervals

When comparing all-comer KM versus 12-lead ECG parameters, the ΔPR was 15.2±10.8 msec (r = 0.86), ΔQRS was 9.6±8 msec (r = 0.86), and ΔQTc was 15.6±12.7 msec (r = 0.83). There was strong correlation between the KM-automated HR and the measured HR on KM (12.5±12.2msec, r = 0.85) as well as when comparing the KM-automated HR and EP-calculated 12-ECG HR (11.2±12 msec, r = 0.87). Perfect agreement (within 20 msec variance) was seen in 28/30 (93%) for heart rate measurements and 114/150 (76%) of total interval measurements: 21/30 PR, 27/30 QRS, 19/30 QT, and 21/30 QTc measurements.

**Fig 2:**
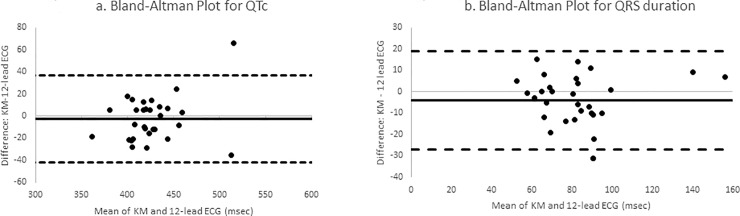
Bland-Altman plots. These Bland Altman plots for QTc (2a) and QRS (2b) demonstrate strong levels of agreement between KM and 12-lead ECG.

KM QTc demonstrated very good agreement (bias = -2.6 msec; standard deviation of bias = 20.2 msec) with the standard 12-lead ECG using the Bland-Altman measurement of agreement. Fig 2(A) demonstrates that the difference in milliseconds of the QTc interval between the KM and 12-lead ECG plotted against the mean of the 2 methods was clinically non-significant.

In patients with absolute mean QTc difference >20 msec, 4/9 (44%) had conduction anomalies (PVCs, PACs, pre-excitation, BBB) and 2/9 (22%) had marked sinus arrhythmia. Only 2 patients (2/30, 6%) had a QTc absolute mean difference >30 msec with both patients having underlying rhythm abnormalities (ventricular pacing; sinus arrhythmia with blocked PAC’s). There was no statistical difference (p = 0.46) in the absolute mean difference in QTc measurement between healthy children and children with underlying CHD/arrhythmia. A QTc interval >450 msec was associated with an increased odds ratio (OR) of not achieving perfect agreement (OR 7.06, 95% CI 1.07–54.09; p = 0.04). Despite this, the sensitivity and specificity for detection of QTc >450 msec were 80% and 96%, respectively. The KM underestimated the QTc in 15 patients (50%) by a mean of 18±8 msec with 10/15 (67%) patients having a diagnosis of a congenital cardiac or conduction anomaly (p = 0.83).

Measurement of the QRS duration also exhibited strong agreement (bias -4.1 msec; standard deviation of bias = 11.9 msec; Fig 2(B)). Perfect agreement for QRS duration was seen in 27/30 (90%) patients. The KM underestimated the QRS duration in 17/30 (57%) patients with a mean absolute difference of underestimation of 12±9msec.

The mean absolute difference and standard deviation for each age subgroup are displayed in Table 2. There was no significant difference between subgroups.

**Table 2:** Variance between Smartphone Monitor and 12-lead ECG parameters. There was no statistical difference in average absolute mean difference for interval measurements between groups.

	Group A (n = 10)	Group B (n = 10)	Group C (n = 10)	p-value
**PR**	12.2 ± 10.7	18.1 ± 9.6	15.5 ± 11.6	0.4
**QRSd**	6.2 ± 5.4	10.1 ± 7	12.4 ± 10.4	0.2
**QT**	22.9 ± 27.6	15.8 ± 12.2	21.9 ± 19.7	0.7
**QTc**	11.2 ± 7	14.1 ± 8.5	21.6 ± 18.3	0.2

### Rhythm assessment

All patients were in sinus rhythm at the time of KM and 12-lead ECG acquisition. There were no patients with sustained arrhythmia. Four patients had isolated premature atrial or ventricular contractions that were captured on either the KM or 12-lead ECG. Due to the serial acquisition of tracings, the isolated premature beats were captured on both modalities in one out of these four patients. Rhythm interpretation on the KM tracing and 12-lead ECG was concordant in 19/30 (63%) paired tracings read by EP#1 and 19/30 (63%) paired tracings read by EP#2 (Table 3). Common discrepancies included normal sinus rhythm versus sinus arrhythmia or sinus tachycardia identified on 4/30 readings by EP#1 and 6/30 readings by EP#2. In 1 patient with ventricular pacing, both overreaders interpreted the KM tracing as sinus rhythm with right bundle branch block versus “A-sensed/V-paced” on standard 12-lead ECG. The KM accurately identified a patient with pre-excitation as well as a patient with 1^st^ degree AV block.

**Table 3:** Rhythm interpretation and comparison by two pediatric electrophysiologists. Abbreviations: SR = sinus rhythm, ST = sinus tachycardia, SB = sinus bradycardia, SAR = sinus arrhythmia, PAC = premature atrial contraction, PVC = premature ventricular contraction, IRBB = incomplete right bundle branch block.

	KM RHYTHM INTERPRETATION			12-LEAD ECG RHYTHM INTERPRETATION	
Patient	KM Automated	EP #1	EP #2	EP #1	EP #2
1	Possible AF	SR	SR with SAR	SR	SR
2		SR	SR	SR	SR
3		SR with long PR	SR with prolonged AV conduction	SB with 1st degree AVB	SB with 1st degree AVB
4		SR with PVCs	SR with PVCs	SR with PVCs in trigeminy and quadrigeminy	SR with frequent PVCs
5		ST	ST	SR	SR
6	Normal	SR with RBBB	SR with RBBB	A sensed/V paced	A sensed/V paced
7		SB with SAR	SB with SAR	SB	SB
8	Normal	SR with SAR; prolonged QT	SR with SA	SR SAR with blocked PACs	SR with blocked PACs
9	Normal	SR	SR	SR	SR
10	Normal	SR	SR	SR	SR
11	Normal	SR with preexcitation	SR with preexcitation	SR, SAR, WPW	SR with WPW
12	Normal	SR	SR	SR with LQTS	SR
13		SR with SAR	SR with SAR	SR SAR	SR with SAR
14	Possible AF	SR	SR	SR	SR
15	Normal	SR	SR	SR	SR with SAR
16	Normal	SR with PVCs	SR with PVCs	SR with SAR	SR
17	Normal	SR SAR	SR	SR	SR
18	Normal	SR SAR	SR with SA	SR	SR
19		SR/ST	SR	ST	SR
20		SR	SR	SR, IRBBB	SR
21	Normal	SR	SR	SR	SR
22		SR	SR	SR SAR	SR with SAR
23		SR	SR	SR	SR
24	Possible AF	SR/ST	SR with SAR	SR SAR	SR
25	Unreadable	SR	SR	SR	SR
26		SR SAR	SR with SAR	SR SAR	SR with SAR
27	Possible AF	ST with PACs with aberrancy	SR with PAC	ST	ST
28		SR/ST	ST	SR	SR
29		SR/SAR	SR with SAR	SR SAR	SR with SAR
30		SR/SAR	SR with SAR	SR SAR	SR with SAR

### Atrial Fibrillation (AF) algorithm

There were no patients with documented AF based on EP interpretations of KM and 12-lead ECG tracings. However, the KM AF algorithm reported 4/30 (13%) false positive “possible AF” diagnoses with EP over-reads reporting sinus tachycardia with sinus arrhythmia (n = 3) and sinus tachycardia with aberrantly conducted PACs (n = 1) resulting in a specificity 87% and negative predictive value of 100%.

## Discussion

This is the first study to directly compare electrocardiographic intervals measured from the KM device to the standard 12-lead ECG in pediatric patients. The data show that the Kardia device can accurately monitor heart rate, rhythm, and interval lengths in children. Healthy children as well as children with underlying congenital heart defects or arrhythmias were included in this study, supporting the generalizability for use of this device in this population. Importantly, all tracings obtained in this study were of diagnostic quality despite 3 patients requiring a second tracing. These diagnostic quality tracings allow providers the ability to remotely treat the identified arrhythmia and triage for further evaluation.

Like others in the literature, this study reveals excellent agreement for heart rate measurement in all age groups. This highlights the potential for the KM device to be used in the outpatient monitoring of heart rate as well as for work-up of palpitations. Age-related compliance has been identified as a barrier to attaining legible ECG data using event-triggered monitoring [8]. The ease of use and accuracy of the KM device makes this an ideal alternative approach for capturing time-sensitive events in young children. These features of the device could also potentially lead to decreased levels of anxiety among these patients who often experience increased levels of stress during diagnostic ECG testing.

Baseline interval agreement was superior to that documented in prior studies. For example, QTc absolute mean difference in this study was 15.6±12.7msec compared to that reported by Haberman et al with mean difference of 33±44 msec in a cohort investigating division I athletes, healthy subjects, and cardiology clinic patients [4]. This is the first direct comparison of QTc intervals specifically in the pediatric population and results for QTc interval measurement are promising when compared to those obtained in the young adult population. The other interval measurements in this study achieved a similar degree of agreement. Our definition of perfect agreement <20msec has been chosen as an aggressive cutoff point in previous studies [6], and the majority of tracings (76%) achieved perfect agreement between the Kardia device and 12-lead ECG.

Absolute mean difference of >20msec was most common in QT and QTc measurement comparisons. Neither age group nor presence of underlying cardiac diagnosis predicted increased differences in absolute mean difference for these intervals. The etiology for bias in these measurements is multifactorial. The Kardia tracings were obtained within 30 minutes of 12-lead ECG acquisition thus leading to the possibility of variability in heart rate and autonomic tone. From direct observation as well as previous literature, the KM is sensitive to motion artifact (muscle tremor, arm movement, muscle tension) and background noise. These parameters are difficult to control for in the pediatric setting. In addition, the average of measurements from two electrophysiologists was used for comparison calculations and, therefore, inter-user variability may have also contributed. Despite these facts, the KM device had a high specificity for detecting a QTc >450 msec and would thus be an effective outpatient tool for monitoring patients with prolonged QTc.

Similarly, QRS duration data exhibited excellent agreement. Although the KM monitor underestimated the QRS duration in the majority of patients, the absolute mean difference in measurements was small (12±9msec) and unlikely to have clinical significance. Thus, the KM monitor would be useful in differentiation of narrow versus wide-complex tachycardias in the outpatient setting.

Agreement in rhythm assessment was seen in the majority of patients. All patients were in sinus rhythm and there were no cases of sustained tachycardia. Ferdman et al has published data supporting the use of the smartphone ECG in diagnosing pediatric SVT, including accuracy in detecting mechanism [9]. Additionally, the specificity of the KM AF algorithm in this study was high. Other studies have documented excellent sensitivity and specificity for detection of AF in the adult population. Our study did not specifically target this group and further studies would be needed to validate the AF algorithm in the pediatric population.

The Kardia mHealth device is an effective and efficient device. Patients and/or caregivers were easily able to follow instructions on how to use the KM monitor. Further usability of the device has been previously analyzed by Nguyen et al who assessed for user satisfaction in pediatric patients with paroxysmal arrhythmias [5]. In that study, 98% of survey responses indicated it was easy to obtain tracings, 93% found it easy to transmit tracings, and 98% showed added comfort in managing their own or their child’s arrhythmia by having the device. The KM’s ease of use combined with its accurate results supports the incorporation of this device into the outpatient management of arrhythmias in children [10].

### Study limitations

There are multiple limitations in this study. First, this is a single center design limiting the generalizability of the results. Additionally, the sample size of 30 patients did not allow for more complex analysis of validity of measurements across age groups or diagnosis. The KM and 12-lead ECGs were obtained serially rather than concurrently leading to potential variability in heart rate and interval measurements. This is certainly a limitation of the study; however, this study design was chosen to attempt to optimize the data quality of tracings obtained from younger patients as concurrent acquisition would have been technically challenging in the infant to early childhood age groups. Lastly, interval measurements were batch read by two electrophysiologists. Although this method of calculation was necessary to account for inter-user variability, it also likely contributed to differences in measurement between the KM and 12-lead ECG.

## Conclusion

The Alivecor Kardia mHealth device produces accurate single lead ECG tracings in both healthy children and children with underlying cardiac disease or rhythm abnormalities across the pediatric spectrum. This mHealth device provides an accurate, non-invasive, and real-time approach for ambulatory ECG monitoring in children and adolescents.

## Abbreviations

KM: Kardia mobile device
EP: electrophysiologist
AF: atrial fibrillation
